# Rochester Pathological Society

**Published:** 1886-03

**Authors:** Edward B. Angell


### ROCHESTER PATHOLOGICAL SOCIETY.

*Meeting of January 28, 1886.*

Reported by EDWARD B. ANGELL, M. D.

DR. W. SIBLEY presented a paper on "The Management of Uncomplicated Labor, with Special Reference to the Methods of Mechanical Interference so much in vogue at the present time." Taking as his text the physiological character of labor in contradistinction to the morbid nature of disease, and as an illustration the report of a case in a recent number of the *Journal of Obstetrics*, the reader made an earnest and able plea for greater patience in waiting on Nature's slower but safer mode of delivery. Prefacing his remarks with a distinct statement that there were many cases of parturition which deviate from the normal condition, such as placenta prævia, threatened eclampsia, faulty pelvis, morbid growths, etc., wherein instrumental means are absolutely required, the essayist condemned, as needless and injurious, interference in those cases which are delayed only through an apparent inability on the part of nature to terminate them successfully. Many reasons often assigned for mechanical interference, such as undilated os, with early rupture of the membranes, hard and rugose vagina, rigid perineum, or suffering of the patient, he considered rather as indications for delay, since they were obstacles which nature would with ease and safety surmount in time.

"It seems to me," the writer said, "that the suffering of a woman in her labor which is physiological, is nothing compared with the suffering and inconvenience of a lacerated os uteri, or a complete rupture of the perineum and their attendant effects, which are pathological. The obstetrical forceps are being used at the present day very extensively. If you ask the physician who makes frequent use of this instrument his reasons, he will very likely tell you, 'O, to save time and relieve the woman from suffering.' When a physician is engaged to attend a case of labor, it is implied that he understands his duty, and will do it. If he accepts the position as attendant, it is certainly his duty to use his best judgment for his patient, and he should not accept that responsibility unless he can devote the time necessary for the completion of labor. His personal interests or comfort should not sway his judgment nor allow him to use means that will be detrimental to his patient's future interests."

Referring to this matter in a recent article, Dr. Frank Hamilton said: "A young man who was urging me to apply the forceps to his patient, though the result proved it unnecessary, told me that a friend of his, a recent graduate of one of the New York schools, and now engaged in a large obstetrical practice in a neighboring town, assured him that he never put a woman to bed without forceps. I do not hold this young man's teachers responsible for his practice, but the fact illustrates what is now the drift of professional opinion. While there can be no question that the forceps and anæsthetics have been of incalculable benefit to parturient women, I have a strong conviction that since they have come into such common and almost universal use they have done a great deal of harm. I believe the time is not far distant in the future when it will become apparent to all that we have in these matters progressed too rapidly, and, possibly, that our progress has been altogether in the wrong direction."

Allow me to call attention to a letter in the *Medical Record*

regarding the methods in vogue at the Vienna Obstetrical Hospital, where the least possible interference with nature in any way is the rule, and strict antiseptic measures rarely adopted. Notwithstanding this reliance upon Nature's weak and tardy efforts, the average mortality is less than one-half of one per cent.

In counting the cost of a forceps delivery, we may look for lacerated os uteri and perineum, abrasions of the mucous membrane, uterine inertia, post partum hemorrhage and septicæmia, in the mother; fracture of the bones, and lacerations or contusions of the soft tissues, facial paralysis, etc., in the child. Furthermore, the accoucheur is unmindful of the pain inflicted on the child whose head he has between the blades of his forceps, during the vigorous effort that is sometimes required in the delivery.

Such, in fact, is the course pursued in many cases where there is not the slightest occasion for such treatment, and it is indeed needful that something should be done to arrest the reckless temerity of men calling themselves physicians, who, if we are to judge them by their acts, place a very insignificant estimate on human life. This may seem very severe language, but I am inclined to believe it is not overdrawn: when we read the reports of cases which, viewed from our standpoint, present symptoms of delayed labor only, but in which the forceps are applied with such fearful results, are we not justified in calling a halt? In my experience of twelve years, in which I have attended about six hundred women, I have never had occasion to use forceps. Neither have I seen, in my own practice, a case of post partum hemorrhage, complete laceration of the perineum, vesico-vaginal or recto-vaginal fistula, and but one case of puerperal fever, and that rather the result of a preceding accident. In this connection, I may add that it has become a routine practice with me to examine carefully all the cases I attend for ruptured perineum.

Post partum vaginal injections are unnecessary and injurious: unnecessary, because nature supplies a protection to the slight abrasions which occur through the plastic lymph thrown out; injurious, because the fluid acid, washing away this protection, will allow the absorption of the uterine discharges. While thus waiting Nature's efforts, much may be done, however, to relieve the discomfort and weariness of the patient through a kind word of encouragement, a change in the position, a little nourishment, or similiar attentions. A close observer of Nature's methods will soon learn that rupture of the membranes often facilitates labor, if they are tough and tenacious. Often the knee-chest position will assist materially in righting a faulty presentation. I well remember a case in which a shoulder presentation was corrected and vertex position gained, followed by easy delivery, through the combined effect of the knee-breast posture and by manual manipulation. With drugs to assist labor I have had very little experience. Ergot I consider potent for good or evil; I never give it except at the close of the second stage. Opium is a remedy of great value. I use it in all stages of labor, if occasion require. Anæsthetics, if used with discretion, are, no doubt, of real service.

Dr. Wm. R. Howard disappoved of the use of forceps, and referred to the well-known fact that a prominent physician of the city had never employed them during the whole of his long experience. He would use no drugs to facilitate labor, but considered the vaginal douche after labor of advantage, because of the comfort it afforded the patient.

Dr. C. M. Briggs had been taught always to carry forceps in his obstetrical practice, but had used them only four or five times. He had used them when he was confident they gave the only hope of delivery. He related a case to which he was called in consultation, in which there had been a delay from impacted occipito-posterior position for three days, with consequent exhaustion. He dilated the os, applied the forceps after placing the patient in the knee-breast posture, thereby relieving somewhat the downward thrust of the head, and delivered the child with safety to both. But, with accumulating experience, he was inclined to be more and more conservative in applying the forceps, greatly preferring the moderate use of chloroform to facilitate labor, especially toward its close. He had found it particularly valuable at that moment of intense suffering when the head presses on the perineum. He recalled one case where the pain was so severe as to cause the patient to relax all effort. The use of chloroform, within the limit of unconsciousness, gave relief, and the child was born at the second or third expulsive effort. He would limit its use in general to those cases where keen, fruitless distress supplants the normal efficient labor pains. Regarding ergot, he usually gave it just previous to the termination of labor.

Dr. Angell called attention to the liability of injuring the head through pressure in the application of the forceps. He referred to two or three cases of severe cerebral disorder under recent observation, wherein the history of each began with an instrumental delivery. A life of disordered intelligence, or incoordinated nervous activity, was a terrible price to pay for the saving of a little time or the sparing of a little suffering. The possibility of disaster to the little life must not be overlooked in the desire to serve the mother.

Dr. Wooden had applied the forceps over one hundred times, and had reached the conclusion that it was wisest to trust more to Nature's measures and use artificial means less. The only feature that required active interference was undilated os, which was best treated by a suppository of half a grain of morphine. That usually gave the patient a brief sleep, and the os was found dilated on awakening. He would do nothing toward rupturing the membranes till the head was pressing well down on the perineum. He never used ergot during labor, inasmuch as, in his experience, before the end of labor, it caused tonic contraction of the uterus, and clonic contractions after, with flowing during the intervals. Regarding the use of the forceps, he believed further that they could not be employed without more or less laceration or bruising of the tissues. He recalled three cases of complete rupture of the perineum, two of which had resulted from the use of forceps, while he remembered several tears extending to the anal sphincter following their application. A three years' record of obstetrical practice had shown him that in his forceps cases he had a higher pulse and temperature, with slower recovery than in his normal labors, while there was a greater tendency to floodings. He had encountered three or four violent post partum hemorrhages, all following the use of forceps.

Dr. Preston referred to the discussion regarding instrumental labor that occurred in the society two years ago. At that time he thought he had used them perhaps too freely, and resolved to leave them at home and send for them only when a necessity arose. The very next case he had was one he had watched carefully during pregnancy, with the anticipation of an uneventful issue. But just as the os was fairly dilated, his patient gave unmistakable signs of impending eclampsia. He sent for his forceps in great haste, delivered safely, and never again went to a case unarmed. It was true that post partum hemorrhage was more frequent in forceps cases, but we must not forget that such cases were usually difficult labors, and therefore more liable to result disastrously. It was furthermore true that all forceps cases get some laceration; he never saw one without some tear, while there was also great danger of abrasions of the mucous surfaces, with consequent risk of septicæmia. He would recommend supporting the protruding arms, but not the advancing head.

Dr. Wm. Moore had applied the forceps forty or fifty times, usually because of exhaustion, but he was using them less frequently as his experience became larger. He advocated the use of opium instead, in order to afford rest.

In reply to a question, Dr. Wooden stated that, in his experience, a patient to whom nourishment was given always vomited before the os was dilated, but never after. Regarding the abdominal bandage, he had abandoned its use for three reasons: 1. It was troublesome to apply. 2. He had noticed the uterus without it was high up in the abdomen, and he feared bad results in consequence of its pressure. 3d. He did not believe it contributed any toward the regaining of form. Its only value consisted in the support it furnished the relaxed abdominal walls.

